# Molecular Characterization of Multidrug Resistant Uropathogenic *E. Coli* Isolates from Jordanian Patients

**DOI:** 10.2174/1874285801812010001

**Published:** 2018-01-31

**Authors:** Yacoub R. Nairoukh, Azmi M. Mahafzah, Amal Irshaid, Asem A. Shehabi

**Affiliations:** Department of Pathology-Microbiology, School of Medicine, The University of Jordan, Amman, Jordan

**Keywords:** Uropathogen, *E.coli*, Multidrug-resistance, ST131 clone, Carbapenemase

## Abstract

**Background:**

Emergence of multi-drug resistant uropathogenic *E. coli* strains is an increasing problem to empirical treatment of urinary tract infections in many countries. This study investigated the magnitude of this problem in Jordan.

**Methods:**

A total of 262 *E. coli* isolates were recovered from urine samples of Jordanian patients which were suspected to have urinary tract infections (UTIs). All isolates were primarily identified by routine biochemical tests and tested for antimicrobial susceptibility by disc diffusion method. Fifty representative Multidrug Resistance (MDR) *E. coli* isolates to 3 or more antibiotic classes were tested for the presence of resistance genes of *blaCTX-M-* 1, 9 and 15, carbapenemase (*blaIMP, blaVIM, blaNDM-1, blaOXA-48*), fluoroquinolones mutated genes (*parC and gyrA*) and clone of ST131 type using PCR methods.

**Results:**

A total of 150/262 (57.3%) of *E. coli* isolates were MDR. Urine samples of hospitalized patients showed significantly more MDR isolates than outpatients. Fifty representative MDR *E. coli* isolates indicated the following molecular characteristics: All were positive for mutated *parC* gene and *gyrA* and for ST131 clone, and 78% were positive for genes of *CTX-M-15*, 76% for *CTX-M-I* and for 8% *CTX-M-9*, respectively. Additionally, all 50 MDR *E. coli* isolates were negative for carbapenemase genes (*blaIMP, blaVIM, blaNDM-1, blaOXA-48*), except of one isolate was positive for *blaKPC-2 .*

**Conclusion:**

This study indicates alarming high rates recovery of MDR uropathogenic *E. coli* from Jordanian patients associated with high rates of positive ST131 clone, fluoroquinolone resistant and important types of blaCTX-M.

## 1 INTRODUCTION

Emergence of *E. coli* strains that produce extended-spectrum beta-lactamases (ESBLs) were increased steadily among commensal and clinical *E. coli* isolates worldwide including Jordan [1-5]. These strains are capable of hydrolyzing penicillins, broad-spectrum cephalosporins and monobactams, but they do not affect the cephamycins or carbapenems and their activity is inhibited by clavulanic acid [6].

Increased occurrence of multidrug resistance (MDR) *E. coli* to 3 or more antibiotic classes causing urinary tract infections will be associated with treatment failure, particularly in association with bacterial strains carrying CTX-M extended-spectrum ESBLs. The genes coding for ESBLs are usually carried by plasmid, which facilitates their spread among most other Gram-negative bacteria. It has been observed in French geriatric hospital that *E. coli* isolates, both CTX-M-type producer and fluoroquinolone-resistant had identical transferable plasmid which may carry other genes responsible for resistance to aminoglycosides, and tetracycline [7]. 

The global spread of carbapenemase-producing *Enterobacteriaceae* constitutes a significant threat for the treatment of patients. In particular, CTX-M-15 is among the commonest CTX-M variants discovered in many genera of *Enterobacteriaceae* across the world, and it has been reported in Europe and Middle East countries [1, 3, 8-10].

A new *E. coli* clone designated sequence type ST131 was found in stool and urine specimens of patients in different countries, and it has been characterized as serotype O25:H4, and it was often associated with fluoroquinolone resistance and CTX-M-15 production [3, 9].

In Jordan, many studies have shown high occurrence of antibiotic resistance in *E. coli* and *K. pneumoniae* clinical isolates associated with the production of ESBLs and CTX-M-groups [1, 3, 11, 12], but to date, there is no single study has attempted to detect the prevalence of ESBLs and fluoroquinolone resistance genes in *E. coli* isolates from urine samples of community-acquired infection or hospitalized patients.

## 2 MATERIALS AND METHODS

### 2.1 Bacterial Strains

This prospective convenience sampling study included *E. coli* isolates from non-repetitive fresh midstream urine (MSU) specimens of 262 patients whom their urine samples were sent to diagnose the cause of UTIs. A total of 227 (86.6%) urine specimens were obtained from outpatients of the Jordan University Hospital laboratory and Biolab (Private Medical Laboratories in Amman), and 35 (13.6%) were recovered from hospitalized patients over the period from March to July, 2016. Relevant data from each patient were obtained and recorded on special form. These included age, gender, name, taking of antibiotic at time of sampling and prior 2 week of sampling. Amman, the capital city of Jordan, has approximately 3.5 million population.

Fresh MSU specimens were collected from each patient using sterile container, then cultured on blood and MacConkey agar plates within 1-2 hours and incubated for 24 hrs at 37°C in each of two the microbiology laboratories. All urine specimens which were positive for *E. coli* counts (>10^5^ CFU/ ml) and showed the presence of at least 10 pus cells/HPF in routine microscopic examination, were considered significant for a suspected UTI case. Five colonies that were morphologically identical to *E. coli* were picked up and subcultured on MacConkey agar to obtain pure *E. coli*. The isolates were primarily identified as *E. coli* by standard biochemical characteristics, including citrate utilization, lactose and glucose fermentation in tubes with Kligler iron agar, urease-negative and indole-positive [1]. All confirmed cultures of *E. coli* isolates were stored at -70ºC in cryotubes that contain brain-heart infusion agar with 15% glycerol. *E. coli* isolates were later used for antimicrobial susceptibility test, DNA extraction and Polymerase Chain Reaction (PCR).

### 2.2 Antimicrobial Susceptibility Testing

All *E. coli* isolates were tested for antibiotic susceptibility according to the recommendation of the Clinical and Laboratory Standards Institute (CLSI, 2014) using the disc diffusion method [13]. Two different types of antimicrobial discs (Oxoid, UK) and Etest strips (Biomerieux, France) were used as shown in Table **1**. *E. coli* ATCC 25922 and *E. coli* ATCC 35218 (A & B-lactamase producer) were used as control strains.

### 2.3 Molecular Tests

Fifty MDR *E.coli* were selected out of 150 MDR isolates to represent their rates of antimicrobial resistance to the number of drug classes as shown in Table **3**. The DNA of 50 representative MDR *E. coli* isolates was extracted by using Wizard® Genomic DNA Purification Kit (Promega, USA), according to the manufacturer manual procedure. PCR was used for the detection of 16 ribosomal RNA (16SrRNA) sequence in MDR *E. coli* isolates as reported by Tsen *et al*. [14]. The bacterial plasmid was extracted using the Zyppy TM Plasmid Miniprep Kit, (Zymo, USA) according to manufactures instructions. Plasmid extraction done for the identification of plasmid encoded carbapenemase genes among MDR *E. coli* isolates as described by the following references: blaOXA-48 [15] blaNDM-1 [16] blaKPC-2 [17]. Also, phylogenetic group I (CTX-M-1), phylogenetic group 9 (CTX-M-9) CTXM 15 and CTX-M 15 were detected as reported by Leflon-Guibout *et al*. [7]. Both blaIMP-15 types and blaVIM -2 types of MBLs as reported by Pitout *et al*. [18], and both mutated fluoroquinolones-resistance genes (*parC*) and (*gyrA*) were detected as reported by Leflon- Guibout *et al*. [7]. Detection of ST131 type was done as described by Clermont *et al*. [10], and a positive *E.coli* control strain was used for both *PbB* and *trpA* genes [3]. The results of five ST131 clone which have represented five groups of MDR *E. coli* isolates (Table **3**) were also compared for *pabB* gene sequence searched on https://www.ncbi.nlm.nih.gov/guide.

### 2.4 Statistical Analysis

Data generated from the study were tabulated as Microsoft Excel sheet and uploaded to Statistical Package for Social Sciences (SPPSS version 20). P ≤ 0.05 was considered statistically significant.

## 3 RESULTS

A total of 262 of patients aged between 1 to 90 years were included in the study. Of these, 50(19.1%) were males and 212 (80.9%) were females. Patients were categorized into four age groups as shown in Table **1**. The second group is the largest with average age of 31.5±8.5 and accounted for 95(36.2%) of patients. No clinical data have been recorded on these patients. The antimicrobial resistance rates among 262 *E. coli* are shown in Table **2**. A total of 150 (57.3%) of *E. coli* isolates were MDR to 3 or more antibiotic classes. The most commonly found drug-resistance combination was for 5 classes of antibiotics (50/150; 33.4%). as shown in Table (**3**). MDR isolates from hospitalized patients were more detected than in outpatients (71.4% *verses* 55.1%), and all patients aged ≥ 45 have significantly more MDR isolates (Table **4**). The minimum inhibitory concentration of MIC_50_ and MIC_90_ of 4 antibiotics among MDR *E. coli* isolates are demonstrated in Table **5**. All MDR *E. coli* isolates were negative for carbapenemase genes (*blaIMP, blaVIM, blaNDM-1, blaOXA-48*) with except of one positive for *blaKPC-2*, but all these isolates were positive for *parC* gene (100%) as shown in Table **6**. Additionally, 78% of the MDR isolates were positive for *genes of CTX-M-15*, 76% *CTX-M-I* and 8% *CTX-M-9*, and all these were also positive for ST131 clone (*PabB* and *trpA* genes) (Table **6**).

## 4 DISCUSSION

Over the last decade, numerous studies in many countries including Jordan have reported generally that *E. coli* causing UTIs is becoming more resistant to antibiotics [9, 19-26]. Therefore, it is important to monitor continuously the antimicrobial susceptibility of uropathogenic *E. coli in vitro* in order to select the proper antibiotic for the treatment of UTIs and to prevent its complications.

The present study has demonstrated that UTIs were mostly associated with females (80.9%) than males (19.1%), and the most common group of Jordanian patients (36.2%) complaining of UTIs were aged between15-45 years. These results are much similar to a previous study published in Jordan 13-year ago [27]. The highest resistance rates among *E. coli* isolates were 69.8%, 57.6% and 57.3%, to nalidixic acid, augmentin, and cortimoxazole, respectively, and these 3 drugs were frequently used in treatment of UTIs over the last five decades in Jordan (Personal communication, Jordan Ministry of Health, Amman). Overall, our results demonstrate that MDR *E. coli* accounted for 57.3% of all isolates, and hospitalized patients carried significantly more MDR *E. coli* to at least 3 antibiotic classes than out patients (71.4% *verses* 54.1%). It is also important to note from our results that all *E .coli* isolates were susceptible to colistin-sulfate and only 2 isolates (1.3%) were resistant to fosfomycin. Both drugs are rarely used in Jordan, especially in treatment of UTIs. Recent studies also from Jordan and Middle East Arab region reported high prevalence of MDR *E. coli* recovered from community and hospitalized patients suffering of UTIs. The rates of MDR *E. coli* in these studies were ranged between 42% and 87.9% [20, 22-26]

There are few studies which had investigated the incidence of CTX-M-types of ESBLs and specifically carbanpenemases among uropathogenic *E. coli* in the Middle East Arab countries [20, 22-24]. This study has found high rates of MDR *E. coli* isolates carried CTX-M-1 (76%) and CTX-M-15 (78%). The occurrence of different CTX-M-groups is variable depending on the geographical regions, but most recent studies have been reported that CTX-M-15 as the most common CTX-M-type -lactamase found in *E.coli* clinical isolates including those from urinary tract infections [8, 23]. The CTX-M enzymes usually have higher activities against cefotaxime than ceftazidime, However, CTX-M-15 can also hydrolyze ceftazidime efficiently [8].

Most *E. coli* isolates in our study had a higher level of resistance to cefuroxime (MIC 19.07 μg/ml) than to ceftazidime (MIC 4.72 μg/ml). In addition, almost all CTX-M positive *E. coli* isolates (90%) were highly resistance to ciprofloxacin with (MIC 2.49 μg/ml) (Table **4**). Much similar results were observed in a recent Jordanian study which has demonstrated 100% resistance to cefuroxime and 95.9% to ceftazidime among all MDR *E. coli* CTX-M-15 producers isolated from feces of infants [1]. However, almost *E. coli* isolates in this study were negative for carbapenemase genes (*IMP*,*VIM*, *NDM-1*, *blaOXA-48*), except one isolates was positive for *KPC-2*. It is important to note her that all *E. coli* CTX-M producers in this study are still susceptible to imipenem as it has been observed in another new combined study from both Jordan and Lebanon [22].

A recent Lebanese study reported that only 16% of *E. coli* isolates from patients with UTIs harbored 4 different ESBL genes (*CTX-M, TEM, SHV, and OXA*) and 14.8% of isolates carried only one enzyme of CTX-M [23]. In general, there is wide variation in distribution of ESBLs and carbapenemase resistance genes from one geographical region to other [27], and it is clear from our results that uropathogenic *E. coli* are mostly producer of ESBLs, but these are still rarely producing carbapenemases. Another important results of this study indicated that all of MDR *E. coli* isolates were positive for ST131 clone in association with resistant to fluoroquinolones. The identity of *E. coli* ST131 was confirmed in five randomly selected isolates for the presence of *pabB* (347 bp) gene and their sequencing has shown 96-99% homology(Sequencing done by Genewiz Company, USA). It has been documented that *E. coli* O25/ST131 strains cause a wide variety of human infections ranging from cystitis to life-threatening sepsis, and both commonly used drugs fluoroquinolones and cortimoxazole are no longer useful for empiric therapy of UTIs [9]. It has also been suggested that the significant rise in ciprofloxacin resistant in community-acquired *E. coli* causing UTIs is due to extensive use of ciprofloxacin in empirical therapy of urinary tract and respiratory tract infections [21]. Additionally, plasmid-mediated quinolone resistance is a major mechanism responsible for increasing this resistance in most enteric bacteria species [28].

## CONCLUSION

This study concludes that high percentage of uropathogenic *E. coli* isolates from outpatients and hospitalized Jordanian patients in Amman area, is multidrug resistant to at least 3 antibiotics, and high rates of isolates harbored CTX-M-ESBL enzymes and ST131 clone in association with resistant to fluoroquinolones.

## Figures and Tables

**Table 1:** Demographic characteristics of 262 examined patients.

Range of Age Groups in Years	Gender		Total No. (%)	Mean± SD
	Male No. (%)	Female No. (%)		
1 - 14	4(1.5)	38(14.5)	42(16.0)	6.4± 3.2
15 - 45	10(3.8)	85(32.4)	95(36.2)	31.5± 8.5
46 - 65	15(5.8)	42(16.1)	57(21.9)	55.5±5.3
>65	21(8.0)	47(17.9)	68(25.9)	75.6±5.4
Total No. (%)	50 (19.1)	212(80.9)	262	–
Mean± SD	55.59±24.6	41.57±24.1	–	–

**Table 2:** Antimicrobial susceptibility pattern of total 262 *E. coli* isolates from urine specimens of both hospitalized and community patients.*

Antibiotics (disk concentration/ug)	No. (%) Resistant
Nalidixic acid (30)	183(69.8)
Cortimoxazole(25)	151(57.6)
Augmentin(30)	150(57.3)
Cefuroxime(30)	130(49.6)
Ciprofloxacin(5)	122(46.6)
Ceftriaxone(30)	112(42.7)
Ceftazidime(30)	88(33.6)
Gentamicin(10)	57(21.8)
Cefoxitin(30)	30(11.5)
Nitrofurantoin(300)	29(11.1)
Fosfomycin (50)**	2(1.3) 0
Colistin sulphate(10)	Null

* A total of 150 (57.3%) of *E. coli* isolates were MDR, and all 35 *E. coli* isolates from hospitalized patients were MDR. ** only the 150 MDR isolates were tested for fosfomycin

**Table 3:** Distribution of 150 MDR *E. coli* isolates to each antibiotic resistant classes and types of antibiotics.

No. and Type of Antibiotic Classes	Type of Antibiotic Resistant in Each Class	No. (%) of MDR *E. coli* Isolates
3 classes of Antibiotics	AUG,NA,ST	23(15.3)
4 classes of Antibiotics	AUG,NA,ST,CXM**	40(26.7)
5 classes of Antibiotics	AUG,NA,ST, CXM, CIP,	50(33.4)
6 classes of Antibiotics	AUG,NA,ST, CXM, CIP, GM	35(23.3)
7 classes of Antibiotics	AUG,NA,ST, CXM, CIP, GM, NI	02(1.3)

*Abbreviation: Augumentin (AUG); Nalidixic acid (NA); Cortimoxazole(TS); Cefuroxime(CXM); Ciprofloxacin(CIP); Gentamicin(GM); Nitrofurantoin(NI)

** Representative of cephalosporin resistance

**Table 4:** Distribution of antimicrobial susceptibility of 262 *E. coli* isolates from community and hospitalized patients according to their age groups.

No. *E. coli* Isolates Community Patients (227)		No. *E. coli* isolates Hospitalized Patients (35)		Range of Age Groups in Years
MDR Isolates	Susceptible Isolates	MDR Isolates	Susceptible Isolates	
15	25	1	1	1 - 14
49	42	2	2	15 - 45
28*	14	12*	3	46 - 65
33*	21	10*	4	>65
125 (55.1)***	102	25 (71.4)**	10	Total no. (%)

* P ≥ 0.05

**Percent of total resistant *E.coli* isolates (P ≤ 0.05), ** Percent of total resistant *E.coli* isolates (P ≥ 0.05)

**Table 5:** Distribution of MICs (μg/ml) among 50 MDR *E.coli* isolates.

Antibiotic	No.(%) Susceptible Isolates	No. (%) Resistance Isolates	MIC_50_	MIC_90_	MIC Range	Breakpoints for Susceptible
Ceftazidime	13 (26)	37 (74)	2.62	4.72	0.016 – 256	≤ 4
Ciprofloxacin	5 (10)	45 (90)	1.38	2.49	0.002 – 32	≤ 1
Imipenem	49 (98)	1 (2)	0.019	0.034	0.002– 32	≤ 1
Cefuroxime	3 (6)	47 (94)	10.59	19.07	0.016 – 256	≤ 4

**Table 6:** Distribution of carbapenemase resistance genes, fluoroquinolones-resistance genes, CTX-M-type genes and ST131 clone among 50 representative MDR *E. coli* isolates.

Type of Detected Genes	No. (%) Positive Isolates
*bla_VIM_*, *bla_IMP_*, *bla_NDM-1_*, *bla_OXA-48,_*	Null
*bla_KPC-2_*	1(2)
*parC and gyrA*	50(100)
*CTX-M-I*	38(76)
*CTX-M-15*	39(78)
*CTX-M-9*	4 (8)
ST131 clone (*pabB* and *trpA*)	50(100)
